# Supplementary material for: WordCommentsAnalyzer: A windows software tool for qualitative research
Source: F1000Res. 2018 Sep 4;7:536. Originally published 2018 May 3. [Version 2] doi: 10.12688/f1000research.14819.2 (PMC6137411; doi:10.12688/f1000research.14819.2)
Supplement: Supplementary file 3 [file f1000research-7-17614-s0002.tgz › b2a79496-4ada-47c4-9682-a99ce867836d.zip!/Eight Iranian Health Journals/Journal of Health System Research _49.docx]

© Journal of Health System Research . This work is licensed under CC BY-NC 4.0 (https://creativecommons.org/licenses/by-nc/4.0/).

Volume & Issue: دوره 6، شماره 1؛ 1389

Title: شيوع لاغري، اضافه وزن و چاقي در دختران دبيرستاني شهر اصفهان

http://hsr.mui.ac.ir/index.php/jhsr/article/view/12

مقدمه: در سال‌هاي اخير چاقي و اضافه وزن در نوجوانان دركشورهاي در حال توسعه و كشورهاي توسعه يافته روند رو به رشدي داشته است. پژوهش حاضر با هدف ارزيابي شيوع لاغري، اضافه وزن و چاقي در دختران دبيرستاني شهر اصفهان انجام شد. روش‌ها: اين مطالعه به صورت توصيفي، تحليلي و مقطعي با نمونه‌گيري طبقه‌اي- تصادفي بر روي 384 دختر دبيرستاني شهر اصفهان در بهار 1385 انجام شد. اطلاعات دموگرافيك و تن‌سنجي توسط يك نفر جمع آوري گرديد. براي تعيين لاغري، اضافه وزن و چاقي داده‌هاي مرجع BMI (Body mass index) براي سن NHANES-1 به كار گرفته شد. یافته ‌ها: بالاترين درصد لاغري (1/14 درصد) در دختران 18 ساله و بالاترين درصد اضافه وزن و چاقي (25 درصد) در دختران 14 ساله ديده شد. به طور كلي 03/7 درصد از دختران لاغر، 1/9 درصد اضافه وزن و 04/1 درصد چاق بودند. نتیجه‌گیری: شيوع اضافه وزن و چاقي در دختران 14 تا 18 ساله شهر اصفهان در مقايسه با ساير شهرهاي كشورمان كمتر بود.

Title: بررسي تأثير آموزش بهداشت به همسران زنان باردار در آگاهي، نگرش و كاهش سزارين‌هاي انتخابي

http://hsr.mui.ac.ir/index.php/jhsr/article/view/13

مقدمه: امروزه آمار و ارقام سزارين در سراسر دنيا و در كشور ما بسيار بالا و روز به روز در حال افزایش است. اين در حالي است كه عمل جراحي سزارين از جمله اعمال جراحي بزرگ محسوب می‌شود كه با عوارض بسيار خطرناك و به ندرت كشنده همراه می‌باشد. مطالعه حاضر با هدف تعيين تأثیر آموزش به همسران زنان باردار در کاهش میزان سزارین‌های انتخابی طراحی شد.  روش‌ها: مطالعه حاضر از نوع كارآزمايي بود که جهت انجام مداخله، 88 زن باردار شكم اول در هفته‌های 28 تا 32 حاملگي مراجعه كننده به مطب‌هاي خصوصي زنان و زايمان شهر اصفهان به طور تصادفي به دو گروه آزمون و شاهد تقسيم شدند. فقط همسران گروه مورد در زمينه زايمان طبيعي و سزارين آموزش ديدند. در ابتدای مطالعه و پس از گذشت 4 هفته، ميزان آگاهي و نگرش هر سه گروه (مادران گروه مورد و همسرانشان و مادران گروه شاهد) سنجيده شد و با پی‌گیری تلفنی نوع زايمان آن‌ها ثبت گرديد. یافته‌ها: مداخله آموزشی در همسران منجر به افزایش معنی‌داری در دانش و نگرش مثبت آنان نسبت به زایمان طبیعی در مادران گروه مورد شد. همچنین، انجام سزارين بدون دليل پزشكي در گروه مورد (5/29 درصد) به طور معني‌داري كمتر از گروه شاهد (50 درصد) بود. نتیجه‌گیری: با توجه به یافته‌های پژوهش چنین استنباط می‌شود که آموزش مردان در افزایش میزان آگاهی، اصلاح نگرش زنانشان و کاهش میزان سزارین‌های انتخابی مؤثر است.

Title: بررسی رفتارهاي پيش‌گيري کننده از ابتلای آنفلونزاي نوع A بر اساس مدل اعتقاد بهداشتی در دانش‌آموزان دبیرستان‌های شهرکرد

http://hsr.mui.ac.ir/index.php/jhsr/article/view/14

مقدمه: بيشترين موارد بيماري ناشي از ويروس آنفلونزای جدید در بين نوجوانان رخ داده است و این نشان از اهمیت این گروه سنی در خصوص انتشار این بیماری و موارد مشابه آن دارد. این مطالعه با هدف بررسی وضعيت رفتارهاي پيش‌گيري کننده از ابتلا و گسترش آنفلونزاي جدید بر اساس مدل اعتقاد بهداشتی در دانش‌آموزان دبیرستان‌های شهرکرد انجام شد. روش‌ها: این مطالعه مقطعی روی 313 دانش‌آموزان دبیرستانی و با استفاده از پرسش‌نامه انجام شد. روایی و پایایی این پرسش‌نامه در مطالعه مقدماتی بررسی و مورد تأیید قرار گرفت (8/0 = α). در تجزیه و تحلیل داده‌ها از نرم‌افزار آماری 15SPSS استفاده شد. یافته‌ها: میانگین سنی دانش‌آموزان مورد مطالعه 31/16 سال بود و 163 نفر از آنان، دانش‌آموزان پسر تشکیل می‌دادند. آگاهی، شدت درک شده و موانع درک شده بیشتر دانش‌آموزان در سطح متوسط قرار داشت. حساسیت درک شده 4/75 درصد دانش‌آموزان و منافع درک شده 6/55 درصد آنان در خصوص آنفلونزاي نوع A در سطح بالایی قرار داشت. همچنین ، رسانه جمعی با بیشترین درصد به عنوان اولین منبع کسب اطلاعات دانش‌آموزان در زمینه آنفلونزا A به دست آمد. نتیجه‌گیری: با توجه به یافته‌های پژوهش و ارتباط سازه‌های تشکیل دهنده مدل اعتقاد بهداشتی با عملکرد دانش‌آموزان، به نظر می‌رسد استفاده از روش‌های مبتنی بر مدل اعتقاد بهداشتی می‌تواند در خصوص رفتار‌های پيش‌گيري کننده از آنفلونزای نوع A مؤثر باشد.

Title: مواجهه با صدا به عنوان عامل خطرساز بیماری‌های قلبی- عروقی کارگران

http://hsr.mui.ac.ir/index.php/jhsr/article/view/15

مقدمه: كارگران‌ در مشاغل‌ مختلف در معرض‌ صدا قرار دارند که در طولانی مدت می‌تواند سبب كاهش‌ شنوايي‌ و اختلال خواب شود. افزايش مواجهه با صدا همچنین می‌تواند در بالا رفتن فشار خون‌، کاهش کارایی و زیاد شدن میزان غیبت از کار تأثیر داشته باشد.  روش‌ها: در اين‌ پژوهش‌ مـورد- شاهدي‌ به تعداد 80 نفر از كارگران‌ یکی از صنایع اصفهان، از نظر مواجهه با صدا و فشار خون مورد مطالعه‌ قرار گرفتند. تراز فشار صوت‌ در خط‌ توليد كارخانه‌ مذكور به روش استاندارد 1996 ISO و با دستگاه‌ صداسنج ‌مدل 440SEL بر حسب دسی‌بل (dBA) اندازه‌گيري‌ شد. در معاینه بالینی در محل کار، فشار خون‌ افراد در گروه‌هاي‌ مواجهه‌ يافته‌ و بدون‌ مواجهه‌ طبق‌ شرايط‌ استاندارد با فشارسنج‌ جيوه‌اي ‌2ALPK بر حسب mmHg اندازه‌گيري‌ شد. جهت‌ تجزيه‌ و تحليل‌ داده‌ها از آزمون‌هاي Independent t و همبستگي‌ Pearson استفاده‌ ‌شد. یافته‌ها: تراز فشار صدا در کارگاه دارای میانگین 56/2 ± 21/95 دسی‌بل بود که به طور معنی‌داری از حد مجاز 85 دسی‌بل بالاتر (2009 ACGIH) می‌باشد (01/0 > P). فشار خون دیاستولی در 8/28 درصد موارد در محدوده طبیعی و در 1/50 درصد موارد در محدوده بالا و در 1/21 درصد موارد بین این دو محدوده قرار داشت. اختلاف میانگین فشار خون سیستولی گروه مورد با گروه  شاهد در سطح 001/0 = P معنی‌دار بود. اما بین میانگین فشار خون دیاستولی این دو گروه اختلاف معنی‌دار نبود. آزمون همبستگی نشانگر وجود رابطه مستقیم و ضعیفی بین فشار خون سیستولی و صدا بود (006/0 > P، 28/0 = r). نتیجه‌گیری: مواجهه طولانی مدت با صدا در محیط کار، در بالا رفتن فشار خون به عنوان عامل خطرساز بیماری‌های قلبی- عروقی نقش دارد. به همین دلیل اجرای برنامه‌های آموزشی برای کارگران و انجام اقدامات کنترلی برای پیش‌گیری از اثرات زیان بار صدا در محیط کار، توجه به فشار خون كارگران‌ و مراقبت ویژه در مورد كارگران با سابقه فشار خون بالا ‌پیشنهاد می‌گردد.

Title: غلظت كادمیوم، كروم، سرب، نیكل و جیوه در سه گونه از ماهیان مصرفی شهر اصفهان

http://hsr.mui.ac.ir/index.php/jhsr/article/view/16

مقدمه: استفاده از منابع خوراكی آبزی، به ویژه ماهیان، به عنوان بخشی از منابع پروتئینی، به علت نیاز روزافزون انسان به غذا افزایش یافته است. بنابراین به موازات افزایش مصرف این منابع، اهمیت بهداشتی ماهیان نیز بیشتر مورد توجه قرار می‌گردد. بررسی فلزهای سمی به علت فرایند تجمع زیستی در بافت موجودات زنده اهمیت زیادی دارد.  روش‌ها: به منظور بررسی غلظت فلزهای سنگین در بافت عضلانی و پوست سه گونه از ماهیان مصرفی شهر اصفهان، تعداد 105 نمونه از ماهیان سرخو، شوریده (ماهیان دریایی جنوب) و كپور پرورشی به طور تصادفی از مراکز عمده فروش ماهی جمع‌آوری شد و مقدار فلزهای سنگین كادمیوم، كروم، سرب، نیكل و جیوه موجود در بافت عضلانی و پوست ماهیان پس از عملیات هضم شیمیایی با دستگاه جذب اتمی و مقدار جیوه به روش فعال‌سازی با نوترون تعیین گردید. یافته‌ها: میانگین غلظت سرب، كروم، كادمیوم، نیكل و جیوه در بافت خوراکی ماهیان سرخو به ترتیب 44/0، 03/0، 06/0، 32/0 و 22/0، در ماهی شوریده به ترتیب 48/0، 06/0، 06/0، 28/0 و 42/0 و در ماهی کپور به ترتیب 48/0، 07/0، 05/0، 3/0 و 22/0 ppm بر حسب وزن خشك ماهی به دست آمد. نتیجه‌گیری: در این بررسی به طور تقریبی، توزیع یكسانی از فلزهای سمی در بافت ماهیان مورد مطالعه وجود داشت، ولی از نظر آماری اختلاف معنی‌داری بین غلظت آن‌ها مشاهده نگردید. میانگین فلزهای سنگین سمی از حد مجاز سازمان جهانی بهداشت بیشتر نبوده است. همچنین نتایج حاصل از انجام این پژوهش نشان داد كه مقدار فلزهای سنگین سرب، كادمیوم، كروم و نیكل به ترتیب در 27، 8، 3 و 25 درصد از نمونه‌های مورد مطالعه بیشتر از حداكثر مجاز سازمان جهانی بهداشت بود.

Title: وجود باکتری اشرشیاکلی در مدفوع خوکچه‌های هندی در کوه کرکس ایران

http://hsr.mui.ac.ir/index.php/jhsr/article/view/18

مقدمه: خوکچه هندی بر عکس جوندگان دیگر، گیاه‌خوار می‌باشد و دستگاه گوارش این حیوان شبیه اسب، خرگوش و چینچیلا است. فلور باکتریایی دستگاه گوارش این حیوان برای هضم گیاهان مناسب است. آگاهی از فلور طبیعی دستگاه گوارش خوکچه هندی از نظر بهداشت عمومی و کاربرد این حیوان در پژوهش‌های آزمایشگاهی بیماری‌های عفونی حایز اهمیت است. در این بررسی نقش باکتری‌های خانواده انتروباکتریاسه‌آ در دستگاه گوارش خوکچه‌های هندی کوه کرکس تعيین گردید. روش‌ها: دو گروه از خوکچه‌های هندی وحشی اطراف پناهگاه کوه‌نوردی کرکس انتخاب شدند و فلورباکتریایی مدفوع آن‌ها از نظر باکتری‌های خانواده آنتروباکتریاسه‌آ با روش‌های باکتری‌شناسی بررسی شد. یافته‌ها: هیچ‌ کدام از نمونه‌های مدفوع جمع‌آوری شده دارای باکتری‌های بیماری‌زای سالمونلا نبود. از مدفوع خوکچه‌های هندی گروه اول، که از بالای پناهگاه کوه‌نوردی انتخاب شدند، باکتری اشرشیا‌کلی جدا نشد. مدفوع خوکچه‌های هندی گروه دوم (پایین پناهگاه کوه‌نوردی) در نوبت اول نمونه‌برداری، 12 درصد و در نوبت دوم تنها 4 درصد دارای باکتری اشرشیا‌کلی بودند. نتیجه‌گیری: اغلب باکتری‌های دستگاه گوارش از طریق غذا و آب آلوده باعث عفونت در انسان می‌شوند؛ در حالی که در این بررسی باکتری‌های فلور طبیعی دستگاه گوارش کوه‌نوردان باعث آلودگی خوکچه‌های هندی به باکتری اشرشیا‌کلی شده و براي مدتي اين باكتري از طريق مدفوع این حیوان دفع شده بود.

Title: مقایسه وضعیت بهداشت محیط مدارس ابتدایی دولتی نواحی مختلف شهر اصفهان* در سال 1387-1386

http://hsr.mui.ac.ir/index.php/jhsr/article/view/20

مقدمه: دانش آموزان حداقل 20 درصد از اوقات خویش را در مدرسه سپری می‌کنند. آموزش و رشد شخصیت آنان به روش‌های آموزشی صحیح و مناسب، فضای فیزیکی مطلوب و محیط روانی مساعد بستگی دارد. دانش آموزان از سرمایه‌های عمده انسانی هر جامعه محسوب می‌گردند. توجه به سلامت و ارتقای بهداشت محیط تحصیلی آنان نوعی سرمایه‌گذاری برای آینده قلمداد می‌گردد. روش‌ها: این مطالعه به صورت توصیفی- تحلیلی انجام شد. تعداد نمونه‌های مورد مطالعه، 77 مدرسه ابتدایی دولتی در شهر اصفهان بود که به صورت تصادفی منظم انتخاب شدند. ابزار گردآوری داده‌ها، فرم ارزیابی بهداشت محیط مدارس بود. این فرم از دو قسمت تشکیل شده است. قسمت اول شامل مشخصات عمومی مدارس و قسمت دوم شامل آیتم‌هایی است که مطلوب بودن قسمت‌های مختلف مدرسه را مورد بررسی قرار می‌دهد. فرم‌های مذکور با مراجعه کارشناس بهداشت عمومی به مدارس، مشاهده و مصاحبه با مدیر مدارس و در مدت 3 ماه تکمیل گردید و سپس مورد تجزیه و تحلیل قرار گرفته، در نواحی مختلف مقایسه به عمل آمد. یافته‌ها: 6/76 درصد از مدارس، دارای وضعیت مطابق با استاندارد و 5/80 درصد از کلاس‌های مدارس دارای وضعیت مطلوب بودند. از نظر وضعیت استانداردهای ساختمانی و کلاس درس در نواحی مختلف، اختلاف معنی‌داری وجود نداشت. وضعیت سرویس‌های بهداشتی (توالت و دستشویی) و آبخوری‌های مدارس در حد مطلوبی بود و اختلاف معنی‌داری از این نظر در بین نواحی وجود نداشت. اما با این حال، ناحیه 1 وضعیت نا‌مطلوب‌تری داشت. 79 درصد از مدارس دارای وضعیت مطلوب از نظر جمع‌آوری و دفع زباله و 49 درصد از مدارس دارای بوفه‌های بهداشتی و مطابق استاندارد بودند. ولی بین نواحی از این دو نظر اختلاف معنی‌داری وجود نداشت. نتیجه‌گیری: به نظر می‌رسد که وضعیت بهداشت محیط مدارس ابتدایی شهر اصفهان به صورت تقریبی در حد مطلوب بوده، اما باید نسبت به بهبود بوفه‌های بهداشتی اقدام گردد که این امر مستلزم توجه بیشتر مسؤولین محترم آموزش و پرورش، متولیان محترم بهداشت و درمان و اختصاص بودجه مناسب جهت حل مشکلات بهداشتی مدارس می‌باشد.

Title: فعالیت‏های فرهنگی ـ تحصیلی دانشجویان دانشکده بهداشت دانشگاه علوم پزشکی اصفهان

http://hsr.mui.ac.ir/index.php/jhsr/article/view/23

مقدمه: جایگاه ویژه دانشگاه در تربیت نسل بالنده انقلاب اسلامی‏و نقش عظیمی‏که دانشگاه در پرورش مدیران و کارگزاران آینده نظام مقدس جمهوری اسلامی ایفا می‏کند، برکسی پوشیده نیست. ضرورت توجه به فعالیت‏های فرهنگی، تحصیلی و اعتقادی دانشجویان به خصوص چگونگی گذراندن اوقات فراغت آن‌ها، در این راستا اهمیت ویژه‏ای دارد. بنابراین لازم است که با راهنمایی این قشر در زمینه برنامه ریزی صحیح برای اوقات فراغت، آنان را در استفاده هر چه بهتر از ایام پرارزش دانشجویی یاری داد. هدف از اجرای این پژوهش شناسایی نقاط قوت و ضعف برنامه‏های تحصیلی دانشجویان بود تا بتوان بر مبنای آن کارآیی برنامه‏ریزی‏های آینده را تقویت نمود.  روش‌ها: این مطالعه به صورت مقطعی بر روی 238 نفر از دانشجویان دانشکده بهداشت انجام گرفت. روش نمونه برداری منظم و تصادفی بود. داده‌ها با استفاده از پرسش‌نامه خود ایفا شامل اطلاعات دموگرافیک، نحوه گذران اوقات فراغت در داخل و خارج از دانشگاه، امور مذهبی، خدمات رفاهی و نیازهای آموزشی دانشجویان جمع‌آوری شد و به وسیله نرم‌افزار SPSS مورد تجزیه و تحلیل قرار گرفت. یافته‌ها: توزیع جنسی دانشجویان مورد مطالعه 4/79 درصد دختر و 6/20 درصد پسر را نشان ‏داد. 1/60 درصد از دانشجویان غیر بومی ‏ ‏بودند و 1/88 درصد از خوابگاه استفاده می‏کردند. 9/84 درصد از دانشجویان مجرد بودند. میانگین و انحراف معیار تعداد ساعات اوقات فراغت دانشجویان در طول شبانه‌روز 2/3 ± 4/6 ساعت بود. 2/31 درصد از دانشجویان هیچگاه در برنامه‏های فرهنگی- مذهبی دانشگاه شرکت نمی‏کردند و 1/12 درصد همیشه در این برنامه‌ها شرکت می‏نمودند. استفاده از کتابخانه در میان این فعالیت‌ها رتبه بالاتری داشت. دانشجویان نیاز به اردوهای یک روزه، برنامه‏های ورزشی و تفریحی، آموزش قرآن و نمایش فیلم را جزء ضرورت‌های خود دانستند. حدود 3/82 درصد از دانشجویان با شعایر مذهبی و اجرای آن موافق بودند. درصد زیادی از دانشجویان از وجود اکثر خدمات رفاهی نظیر وام تحصیلی و مشاوره روانی بی‌اطلاع بودند. اولویت نیاز دانشجویان برای آموزش گرفتن مواردی نظیر روش‌های جستجوی مقالات، استفاده مناسب از کامپیوتر، نقش استاداند راهنما در حل مشکلات آموزشی و کسب مهارت‌های لازم در پر کردن اوقات فراغت آن‌ها بود. نتیجه‌گیری: از آن جایی که اکثر دانشجویان مجرد بوده، بیشتر وقت خود را در خوابگاه و دانشگاه سپری می‏کنند، لازم است مسؤولان دانشگاه برنامه ریزی دقیقی برای پر کردن اوقات فراغت دانشجویان انجام دهند. توجه به عمده‏ترین نیازهای دانشجویان از قبیل کامل بودن کتابخانه‌ها، اردوهای یک روزه، برنامه ورزشی، مراسم به مناسبت‏های مختلف در ایام سال و آموزش قرآن کریم حایز اهمیت زیادی می‏باشد.

Title: ارزیابی‌ ايستگاه‌هاي‌ كار يك‌ شركت‌ صنايع‌ فلزي‌ با استفاده از روش‌ فنـلاندي‌

http://hsr.mui.ac.ir/index.php/jhsr/article/view/24

مقدمه: ایستگاه کار شامل محیط فیزیکی اطراف کارگر، ابعاد و نحوه چیدمان تمام تجهیزات است. افزايش‌ فشار كار سبب‌ كاهش‌ كارآيي‌ و بازده‌ نيروي‌ كار و در نتيجه‌ افت‌ كيـفيت‌ محـصولات‌ توليدي‌ خواهد شد. با شناخت‌ عوامل‌ مؤثر در افزايـش‌ فشار كار و كاهش‌ يا بر طرف‌ كردن‌ اين‌ فـشارهاي‌ تهديد كننده‌ سلامتي‌، علاوه‌ برحفظ سلامت کارگر، از زيانـ‌هاي‌ اقتـصادي‌ نيز جلوگيري‌ مي‌شود. هدف‌ از اين‌ پژوهش تعیین، ارزیابی وضعيت‌ ايـستگاه‌هاي‌كاري‌ از نظر عوامل‌ انساني‌، شغلي‌ و محيطي‌ زمينه‌ساز حوادث‌ و بيماري‌هاي‌ شغلي‌ با استفاده از روش فنلاندی بود. روش‌ها: اين‌ مطالعه مقطعي،‌ به‌ روش‌ سرشماري‌ در تمام ايستگاه‌هاي‌ كار  يك‌ شركت‌ صنـايع‌ فلزي‌ اصفهان انجام‌ شد. اطلاعات‌ مورد نظر با استفاده‌ از چـك‌ ليست‌ استاندارد فنلاندی آنالیز ارگونومی‏ محل کار و از طريق‌ مشاهده‌ عيني ‌جمع‌آوري‌ گردید. سپس اطلاعات با استفاده از نرم‌افزار آماری SPSS مورد تجزیه و تحلیل قرار گرفت. یافته‌ها: در 7/84 درصد موارد هيچ‌ يك‌ از استانداردهاي‌ وضعيت‌ فضاي‌ كار در ايستگاه‌هاي‌ كار نبود؛ در 6/13 درصد نحوه‌ فعاليت‌ بدني‌ و در 3/15 درصد بلندكردن‌ بار نامناسب‌ بود؛ در 1/5 درصد مقدار فشار اعمال‌ شده‌ به‌ بازوها بسيار شديد و وضع بدن در حين‌ كار سنگين‌ بد بود؛ در 7/23 درصد احتمال‌ وقوع‌ حادثه‌ زیاد بود؛ در 5/52 درصد به‌ دليل‌ وضعيت‌ كار، امكان‌ ارتباط‌ با ساير افراد محدود شده بود؛ در 8/89 درصد مشکلی در تصمیم گیری وجود نداشت؛ در 1/33 درصد طول‌ سيكل‌ كار كمتر از پنج ‌دقيقه تکراری بود و در 44 درصد موارد روشنایي‌ كمتر از حد مجاز بود؛ شاخص‌ استرس گرمایي‌ حداكثر 29 درجه‌ سانتي‌گراد و زمان‌ مواجهه‌ كمتر از حد اسـتانـدارد بود و استرس‌ گرمايي‌ وجود نداشت. صدا در 20 درصد موارد بالاتر از حد مجاز بود. نتیجه‌گیری: نامناسب‌ بودن‌ محيط‌ كار مشكلاتي‌ براي‌ تحقق‌ وظايف‌ كار شاغلان‌، سلامتي‌ آن‌ها و كـيفيـت‌ محصولات‌ شركت ‌به وجود مي‌آورد. وضعيت‌ فضاي‌ كار، شرايط‌ محيطي‌ و طرز قرار گرفتن‌ بدن‌ در هنگام‌ كار، زمينه‌ ساز حوادث‌ و بيماري‌هاي‌ شغلي‌ است‌. نتايج‌ مي‌تواند مورد استفاده‌ مهندسان‌ طراح‌ شركته‌اي‌ صنايع‌ فلزي‌ مشابه‌ قرار گيرد و می‏توان با طراحي‌ صحيح‌ ايستگاه‌ كار، تا حد زيادي‌ از اين‌ فشارهاي‌ تهديد كننده‌ سلامتي‌ كارگران‌ جلوگيري‌ نمود.

Title: بررسي ارتباط قد و وزن كودكان بدو ورود به دبستان با وضعيت دريافت وعده‌هاي غذايي و ميزان تحصيلات والدين

http://hsr.mui.ac.ir/index.php/jhsr/article/view/26

مقدمه: بيش از نيمي از مرگ‌هاي كودكان ممكن است به طور مستقيم و غيرمستقيم به علت فقر تغذيه‌اي باشد. بنا بر اعلام يونيسف، اهميت نسبي علل زمينه‌اي سوء تغذيه بايد به طور وسيعي در خانواده‌ها، جمعيت‌ها وكشورها شناخته شود؛ این سازمان ت‍أكيد مي‌كند که براي بررسي بيشتر علل سوء تغذيه، يك برخورد كاملاً محلي و ناحيه‌اي، به جاي راه‌حل‌هاي‌كشوري يا جهاني مورد نياز است. هدف از اين مطالعه، تعيين وجود ارتباط بين سطح تحصيلات والدين كودكان بدو ورود به دبستان و وضعيت دريافت وعده‌هاي غذايي اين كودكان با قد و وزن آن‌ها بود. روش‌ها: در اين پژوهش توصيفي- مقطعي، 235 دانش آموز بدو ورود به دبستان در شهرستان خمین، كه در طرح سنجش سلامت كودكان ‌پيش‌دبستاني شركت كرده ‌بودند، به روش نمونه گيري تصادفي سيستماتيك در شهر و روش نمونه گيري خوشه‌اي در روستا انتخاب شدند. به كمك پرسش‌نامه‌، اطلاعات مربوط به تحصيلات والدين و وضعيت دريافت وعده‌هاي غذايي كودكان جمع آوري شد. اندازه گيري قد و وزن توسط پزشك مسؤول طرح صورت گرفت. اطلاعات به دست آمده توسط آزمون‌هاي آماري t مستقل، 2χ، همبستگي Spearman، Mann-Whitney و آناليز كواريانس تجزيه و تحليل شد. یافته‌ها: حدود 58 درصد كودكان دريافت وعده‌هاي غذايي نامناسب داشتند، كه از اين نظر بين پسرها و دخترها و نيز در شهر و روستا تفاوت معني‌داري وجود نداشت (221/0 = P و 568/0 = P). قد و وزن كودكاني كه والدينشان تحصيلات بالاتري داشتند، نسبت به ‌كودكاني كه والدينشان تحصيلات پایين‏تري داشتند، بيشتر بود (29/0 = rs براي قد و 31/0 = rs براي وزن در مادران، 01/0 > P). ميانگين قد و وزن كودكاني كه دريافت وعده‌هاي غذايي مناسبي داشتند، نسبت به ميانگين قد و وزن كودكاني كه دريافت وعده‌هاي غذايي مناسبي نداشتند، به طور معني‌داري بالاتر بود (001/0 > P). نسبت وزن به قد در كودكاني كه والدينشان تحصيلات بالاتري داشتند، نسبت به كودكاني كه والدينشان تحصيلات پايين‌تري داشتند، بيشتر بود (28/0 = rs براي تحصيلات مادر و 27/0 = rs براي تحصيلات پدر، 01/0 > P). نتیجه‌گیری: به نظر مي‏رسد، بازنگري جدي در برنامه تغذيه‌اي گروه سني پيش دبستاني و ارایه الگوهاي صحيح تغذيه‌اي براي اين گروه ضروري ‌باشد. از طرفي، طراحي و اجراي برنامه‌هاي آموزشي مرتبط با سلامت كودكان براي خانواده‌ها و به ويژه مادران، بايد بيش از پيش مد نظر مديران و برنامه ريزان بخش سلامت و آموزش‌كشور قرار گيرد. واژه‌های کلیدی: .

Title: بررسي تأثير آموزش بر آگاهي و نگرش تغذیه‌ای دانش آموزان دختر دبيرستاني

http://hsr.mui.ac.ir/index.php/jhsr/article/view/144

مقدمه: با توجه به این که دختران نوجوان امروز، مادران فردا خواهند بود، این دوران یک فرصت طلایی برای ارتقای آگاهی‌های تغذیه‌ای نوجوانان در جهت بهبود سلامت نسل آینده می‌باشد که با اجرای برنامه‌های آموزشی به عنوان راهبردی مؤثر در جهت بهبود الگوهای غذایی این سنین میسر می‌گردد. مطالعه حاضر با هدف بررسی تأثیر آموزش تغذیه بر آگاهی و نگرش دانش آموزان دختر دبیرستانی انجام گرفت. روش‌ها: در این مطالعه 315 نفر از دانش آموزان دختر 9 مدرسه از نواحی مختلف اصفهان در دو گروه تقسیم شدند. قبل از مرحله اول آموزش و 10 روز بعد از جلسه دوم آموزش، به وسیله دو پرسش‌نامه آگاهی و نگرش اطلاعات جمع‌آوری شد. آموزش به صورت ارایه سخنرانی، ارایه جزوات و پمفلت طی 2 جلسه 2 ساعته با فاصله 10 روز اجرا شد و به گروه شاهد نیز آموزش داده نشد. اطلاعات حاصل به وسیله نرم‌افزار 16 SPSSتجزیه و تحلیل شد. یافته‌ها: قبل از اجرای آموزش، گروه‌ها با هم اختلاف معنی‌داری نداشتند ولی پس از مداخله، سطح آگاهی در گروه مورد به صورت معنی‌داری افزایش یافت (0001/0 > P). نگرش در هیچ یک از گروه‌ها تغییر معنی‌داری نداشت. نتیجه‌گیری: از آن جایی که آموزش تغذیه راهکاری با اثربخشی مطلوب در جهت ارتقای آگاهی‌های تغذیه‌ای به شمار می‌آید، مطالعه حاضر نشان داد که با طراحی یک برنامه جذاب آموزشی می‌توان آگاهی‌های تغذیه‌ای دختران نوجوان را به میزان قابل توجهی ارتقا داد. اگر آموزش‌ها با نیازهای تغذیه‌ای این سنین منطبق و به شکل قابل لمس با استفاده از فن‌آوری سمعی- بصری ارایه گردد، اثربخشی نه تنها در آگاهی‌های آنان بلکه در اصلاح الگوی رفتارهای غذایی آنان نیز پدیدار خواهد شد.

Title: سزارین و عوامل مؤثر بر آن در بيمارستان‌هاي دولتي و خصوصي شهر اصفهان

http://hsr.mui.ac.ir/index.php/jhsr/article/view/28

مقدمه: به علت خطرات سزارين نسبت به زايمان طبيعي و عوارض زودرس و ديررس سزارين، اين عمل نسبت به زايمان طبيعي از نظر بهداشت عمومي مورد توجه خاصي قرار گرفته است. هدف از انجام اين پژوهش، تعیین فراواني نسبي سزارين و علل آن بود تا بر اساس نتايج به دست آمده در جهت كاهش ميزان سزارين‌هاي غير ضروري برنامه‌ريزي شود.  روش‌ها: در طول یک سال، 360 نمونه از بين كليه‌ زناني كه در 4 بیمارستان دولتی و 4 بيمارستان خصوصی شهر اصفهان زايمان كرده بودند، به طور تصادفي انتخاب شدند و چك ليست مربوط با توجه به پرونده‌هاي بيمارستاني تكميل گرديد. داده‌ها به روش آمار توصیفی و آزمون‌های آماری مورد تجزيه و تحليل قرار گرفت. یافته‌ها: 6/73 درصد از مادران به روش سزارين و 4/26 درصد به روش طبيعي زايمان كرده بودند که معادل 8/2 سزارين به ازای يك زايمان ‏بود. بيشتر مادران سزارين شده، در گروه سني 29-25 سال و اكثر مادران سزارین شده نخست‌زا بودند. تمايل مادران مراجعه كننده به بيمارستان‌هاي خصوصي جهت انجام سزارين، بيشتر از مراجعين به بيمارستان‌هاي دولتي بود. شايع‌ترين علل سزارين، سزارين قبلي، زايمان سخت، نماي بريچ، سزارين انتخابي و زجر جنيني بود. نتیجه‌گیری: با توجه به آمار بالاي سزارين در بيمارستان‌هاي مورد مطالعه‌ شهر اصفهان (6/73 درصد) در مقايسه با ميانگين استاني آن (5/59 درصد)، ضروري است در زمينه‌ كاهش سزارين برنامه‌ريزي‌هاي لازم صورت گيرد و با افزايش آگاهي زنان و حتي همسران آن‌ها نسبت به روش‌هاي زايماني، از ميزان سزارين‌هاي انتخابي كاسته شود. توصيه مي‌شود بيشتر برنامه‌ها روي زنان نخست‌زا متمركز گردد.

Title: شناخت برخی از عوامل خطر خودکشی و اقدام به خودکشی در استان ایلام و جهت عملکرد آن‌ها

http://hsr.mui.ac.ir/index.php/jhsr/article/view/33

مقدمه: خودکشی و اقدام به خودکشی پدیده‌های پیچیده روانی هستند که تحت تأثیر متقابل عوامل فردی و محیطی قرار دارند. از آن جایی که شناخت عوامل خطر مربوط به هر پدیده از اهمیت بسزایی برخوردار می‌باشد، هدف مطالعه حاضر شناخت مهم‌ترین عوامل خطر اين دو پديده و جهت عملکرد هر یک از آن‌ها در استان ایلام بود. روش‌ها: نظام جامع ثبت اطلاعات موارد خودکشی و اقدام به خودکشی در استان ایلام که در فاصله سال‌های 1375 تا 1382 فعالیت داشته است، داده‌های مطالعه حاضر را فراهم نموده است. اطلاعات مربوط به موارد خودکشی و اقدام به خودکشی رخ داده در استان با ذکر مشخصات دموگرافیک هر مورد شامل سن، جنس و وضعیت تأهل همراه با روش خودكشي، مكان و زمان رخداد حادثه، در این نظام جامع ثبت ‌شده است. یافته‌ها: يافته‌ها نشان مي‌دهد كه اقدام در فصل بهار، استفاده از روش دار زدن، اسلحه گرم و خودسوزي به طور معني‌داري باعث افزايش و متأهل بودن، در گروه سني 40-49 سال قرار داشتن، استفاده از سم و قرص به طور معني‌داري باعث كاهش احتمال ارتكاب به خودكشي در مقايسه با اقدام به خودكشي مي‌شود. نتیجه‌گیری: مراكز بهداشتی و درمانی موجود در استان ايلام به منظور كاهش ميزان خودكشي بايستي به پيشگيري مرحله اول خودكشي به ويژه در مواردي كه در افراد مجرد و يا در فصل بهار رخ داده است و يا از روش‌هاي بسيار كشنده‌ايي نظير دار زدن، اسلحه گرم و خودسوزي استفاده كرده‌اند توجه بيشتر معطوف نمايند.

Title: شکاف کیفیت خدمات بهداشتی اولیه ارایه شده در مراکز بهداشتی درمانی شهر اصفهان از دیدگاه مادران مراجعه کننده به این مراکز

http://hsr.mui.ac.ir/index.php/jhsr/article/view/34

مقدمه: شکاف کیفیت خدمات، فاصله بین ادراکات و انتظارات مشتریان از خدمات ارایه شده می‌باشد. گام اساسی در حذف این شکاف، شناسایی ادراکات و انتظارات مشتریان از کیفیت خدمات ارایه شده است. هدف از اين مطالعه نیز تعيين ديدگاه زنان از شکاف کيفيت خدمات بهداشتي اوليه در مراکز بهداشتي درماني شهر اصفهان بود. روش‌ها: اين مطالعه به صورت مقطعي انجام شد و جمعيت مورد مطالعه آن، زنان مراجعه کننده به مراکز بهداشتي درماني شهری اصفهان بودند. حجم نمونه 1280 نفر بود و برای جمع آوری اطلاعات نیز از ابزار Service quality استفاده شد که شامل ابعاد ملموس، اطمینان، پاسخ‌گویی، تضمین و همدلی در نحوه ارایه خدمت است. اطلاعات به دست آمده با استفاده از آزمون t و 2χ مورد تجزیه و تحلیل قرار گرفت. یافته‌ها: در هر 5 بعد کیفیت خدمات، زنان اختلاف نظر داشتند. کمترین میانگین اختلاف کیفیت (08/11-) در بعد تضمین و بیشترین میانگین آن (41/14-) در بعد ملموس به دست آمد؛ تفاوت مشاهده شده بین نظر زنان در مورد کیفیت خدمات در هر 5 بعد معنی‌دار بود (05/0 > P). نتیجه‌گیری: اختلاف منفي، بيانگر اين است که انتظارات خدمت گيرندگان فراتر از ادراکات آن‌ها از وضعيت موجود بوده، هنوز فضاي زيادي براي بهبودي و اصلاح به منظور نيل به رضايت مشتريان و ارایه خدمات بهداشتي درماني مطلوب وجود دارد.

Title: نگرش دانشجویان دانشکده بهداشت دانشگاه علوم پزشکی اصفهان نسبت به رشته تحصیلی و آینده شغلی خود

http://hsr.mui.ac.ir/index.php/jhsr/article/view/35

مقدمه: دانشگاه‌ها منشأ تحولات جامعه در عرصه‌های مختلف است و دانشجویان به عنوان ارگان اصلی دانشگاه در آینده پیکره اساسی سازمان‌ها و ارگان‌های مختلف جامعه را تشکیل می‌دهد. بنابراین نگرش دانشجویان به رشته تحصیلی و آینده شغلی آن‌ها و پاره‌ای از عوامل مرتبط با تحصیل از جمله جنس، سن، رشته تحصیلی و ... که خود عامل مؤثری در ایجاد انگیزش آن‌ها می‌باشد، باید مورد بررسی قرار گیرد. این مطالعه با هدف تعیین نگرش دانشجویان دانشکده بهداشت دانشگاه علوم پزشکی اصفهان نسبت به رشته تحصیلی و آینده شغلی و پاره‌ای از عوامل مرتبط با آن صورت گرفت.  روش‌ها: در این مطالعه توصیفی- مقطعی، 318 نفر از دانشجویان دانشکده بهداشت مشغول به تحصیل در 4 رشته بهداشت عمومی، بهداشت محیط، بهداشت حرفه‌ای و تغذیه به طور تصادفی انتخاب شدند. ابزار جمع آوری اطلاعات، پرسش‌نامه محقق ساخته بود که با 44 سؤال نگرش فرد نسبت به رشته تحصیلی و آینده حرفه‌ای خود از لحاظ تأمین اقتصادی، تأمین شغلی، رفاه شخصی و رضایت قلبی و مهم‌ترین مشکلات آن‌ها را مورد بررسی قرار می‌داد. داده‌ها به صورت توصیفی و تحلیلی در نرم افزار SPSS تجزیه و تحلیل شد. یافته‌ها: میانگین و انحراف معیار نمره نگرش دانشجویان نسبت به رشته تحصیلی خود 008/7 ± 25/61 به دست آمد. 6/95 درصد از دانشجویان به رشته تحصیلی خود نگرش مثبت داشتند. میانگین و انحراف معیار نمره نگرش دانشجویان به آینده شغلی خود 36/10 ± 54/62 به دست آمد. دیدگاه 4/87 درصد از دانشجویان به آینده شغلی مثبت بود. مقایسه نمره نگرش افراد بر حسب سن و میانگین معدل دیپلم اختلاف معنی‌داری را نشان نداد. در حالی که مقایسه نمره نگرش افراد بر حسب رشته تحصیلی و نحوه نگرش به رشته تحصیلی اختلاف معنی‌داری را با نگرش به آینده شغلی نشان داد. علاقه فردی، خدمت به جامعه، جایگاه اجتماعی رشته و تلقین جامعه بر اهمیت رشته از مهم‌ترین عوامل انگیزش مثبت به شغل بودند. نتیجه‌گیری: با توجه به نتایج حاصل از این پژوهش، دانشجویان رشته‌های بهداشتی نسبت به رشته تحصیلی و آینده شغلی خود نگرش مثبتی داشتند. توزیع مناسب نیروی انسانی، حمایت مالی از استخدام شدگان، ایجاد مراکز مشاوره و حمایت از دانشجویان در توجیه آینده شغلی می‌تواند در تقویت نگرش آن‌ها مفید واقع شود.

Title: مقایسه نیازهای بهداشتی درمانی دهان و دندان سالمندان مقیم و غیر مقیم در خانه سالمندان شهرستان یزد

http://hsr.mui.ac.ir/index.php/jhsr/article/view/57

مقدمه: کهولت سن پدیده‌ای است که تحقق آن مستلزم وجود شرایط مناسب زیستی است. بر طبق گزارش سازمان بهداشت جهانی در سال 2001 تعداد افراد بالای 70 سال به مرز 585 میلیون نفر رسیده، که 9 درصد جمعیت جهان را در بر می‌گیرد و پیامد آن مشکلات مربوط به مراقبت، نگه‌داری، بهداشت و تأمین مایحتاج سالمندان خواهد بود؛ به طوری که مشکلات دهان و دندان یکی از مسایل اولیه در میان سالمندان است. هدف از انجام این تحقیق تعیین نیازهای بهداشتی درمانی دهان و دندان در سالمندان بود. روش‌ها: این مطالعه با روش توصیفی- مقطعی به منظور مقایسه نیازهای سالمندان مقیم و غیر مقیم در خانه سالمندان انجام شد. 77 نفر گروه اول از ساکنین خانه سالمندان و 74 نفر گروه دوم از افراد غیر مقیم، بر طبق پرسش‌نامه از قبل تعیین شده، مورد معاینه و بررسی قرار گرفتند و اطلاعات با استفاده از نرم‌افزار SPSS و آزمون 2χ مورد تجزیه و تحلیل آماری قرار گرفت. یافته‌ها: دو گروه در متغیر مشکلات دهان و دندان اختلاف معنی‌داری با یکدیگر نداشتند (6/0 = P)، ولی از نظر متغیرهای تمایل به درمان (0001/0 = P)، نیاز به درمان (0001/0 = P)، پوسیدگی دندان (049/0 = P)، وضعیت بی‌‌دندانی (009/0 = P)، دندان مصنوعی (0001/0 = P) و دفعات مسواک زدن در روز (0001/0 = P) بین گروه غیر مقیم و مقیم اختلاف معنی‌دار مشاهده گردید. نتیجه‌گیری: با توجه به نتایج به دست آمده، هر دو گروه نیازمند به توجه بیشتر به وضعیت سلامت دهان و دندان می‌باشند و این امر ضرورت ارتقای مراقبت‌های بهداشتی دهان را هر چه بیشتر آشكار می‌سازد.

Title: تأثير دستگيره ضد ارتعاش بر میزان ارتعاش تولید شده توسط دستگاه سنگ سمباده

http://hsr.mui.ac.ir/index.php/jhsr/article/view/22

مقدمه: ارتعاش دست- بازو نوعی ارتعاش موضعی است که اندام فوقانی را درگیر می‏کند. پیامد‏های ناشی از این نوع ارتعاش شامل اختلالات عروقی، عصبی و اسکلتی- عضلانی می‌باشد. هدف از انجام این پژوهش، تعیین تأثیر دستگیره ضد ارتعاش ساخته شده توسط پژوهشگران بر فراوانی اختلالات عروقی و عصبی در کاربران سنگ سمباده‏ دستی بود. روش‌ها: این مطالعه بر روی 12 نفر از کاربران سنگ سمباده‏ دستی در یکی از صنایع فلزي مربوط به توليد ماشين آلات خدمات شهري اصفهان انجام شد. نوع مطالعه تجربی و به صورت قبل- بعد بود. متغيرهاي باليني مورد بررسي شامل وجود یا عدم وجود بی‌حسی، سوزن سوزن شدن، رنگ پریدگی، خارش، ضعف، سردی، درد، کبودی و قرمزی در انگشتان یا دست بود که با استفاده از چک لیست و معاینه بررسی شد. اندازه‏گیری ارتعاش در 3 محور x، y و z مطابق با استاندارد 2003-5349 ISO انجام گرديد. دستگیره ضد ارتعاش با توجه به وزن و فرکانس دستگاه سنگ سمباده طراحی و در مجموع از 9 قطعه مجزا ساخته شد. وزن این دستگیره 214 گرم بود. دستگیره به مدت یک هفته و به طور ميانگين 3 ساعت (± 20 دقیقه) در يك نوبت کاری توسط کاربران مورد استفاده قرار گرفت. قبل و يك هفته بعد از استفاده از دستگيره ضد ارتعاش، كارگران از نظر باليني ‏و چک لیست مورد ارزيابي قرار گرفتند. نتایج کیفی و کمی‏ به ترتیب با آزمون‌های مک نمار و Paired t و با استفاده از نرم‌افزار SPSS مورد تحلیل قرار گرفت. یافته‌ها: در آزمون مک نمار برای متغيرهاي باليني بی‌حسی 01/0 > P، سوزن سوزن شدن 05/0 > P، رنگ پریدگی 01/0 > P، خارش 005/0 > P و درد 005/0 > P، به دست آمد که اختلاف معنی‌داری را نشان می‏داد. مقدار میانگین شتاب ارتعاش قبل از مداخله در سه جهت x، y و z به ترتیب m/s2 6/2، m/s2 8/3 و m/s2 4/3 بود که بعد از مداخله به m/s2 2/1، m/s2 1/2 و m/s2 8/1 کاهش یافت. بیشترین میزان کاهش موردي در محور z و به ميزان 90 درصد مشاهده گردید. کاهش شتاب ارتعاش و رساندن آن به زیر m/s2 2 باعث کاهش اختلالات عروقی و عصبی گرديد. پس از يك هفته استفاده از دستگيره‏ ضد ارتعاش، علایم و شكايات باليني به ميزان ثابت رسيد. نتیجه‌گیری: به طور کلی مقادیر به دست آمده نشان دهنده تأثیر مثبت دستگیره‏ ضد ارتعاش در کاهش میزان اختلالات عروقی و عصبی و همچنین کاهش میزان ارتعاش انتقال یافته به دست می‏باشد. پژوهشگران استفاده از دستگيره‏هاي ضد ارتعاش با ميزان ميرايي ارتعاش به زير m/s2 2 را جهت كاهش عوارض عصبي عضلاني ناشي از ارتعاش دست- بازو پيشنهاد مي‌كنند.

Title: بررسی علل در آوردن IUD و رابطه آن با برخی از عوامل زمینه‌ای در زنان

http://hsr.mui.ac.ir/index.php/jhsr/article/view/56

مقدمه: شناخت عللی كه سبب قطع استفاده از IUD می‌شود، برای برنامه‌ریزی كنترل جمعیت از اهمیت ویژه‌ای برخوردار است؛ این مسأله، انجام بررسی دوره‌ای عوامل مؤثر بر كاهش استمرار IUD و عوارض منجر به قطع مصرف آن را در منطقه خاص توجیه می‌نماید. پژوهش حاضر به منظور بررسی فراوانی نسبی در آوردن IUD به علل مختلف در زنان انجام گرفت. روش‌ها: بررسی حاضر یك مطالعه توصیفی‌ـ تحلیلی و جمعیت مورد مطالعه زنان مراجعه كننده به مراكز بهداشتی درمانی شهرستان لنجان اصفهان جهت خروج IUD بودند. نمونه مورد مطالعه شامل 150 نفر از زنان جمعیت فوق بود كه به روش انتساب متناسب با جمعیت هر مركز به صورت تصادفی طبقه‌ای انتخاب شدند. یافته‌ها: از كل زنان مورد مطالعه، 3/71 درصد داوطلبانه اقدام به در آوردن IUD كرده بودند و در 7/28 درصد IUD خود به خود در آمده بود. شایع‌ترین علت در آوردن IUD در زنان مورد مطالعه، خون‌ریزی بود كه 38 درصد از مراجعین را تشكیل می‌داد. دفع خود به خود IUD در 7/28 درصد، تمایل به حاملگی در 7/14 درصد و عفونت در 10 درصد به ترتیب در مرتبه‌های بعد قرار داشت. سایر موارد 8/8 درصد كل زنان را تشكیل می‌داد. نتیجه‌گیری: علاوه بر انجام مشاوره دقیق، آموزش صحیح، مراجعه مكرر و منظم برای كنترل IUD و تكنیك گذاشتن IUD به طور استریل و صحیح و نیز مسؤولیت‌‌پذیری و مهارت پرسنل مورد تأكید این مطالعه بود كه توجه بیشتر مسؤولین را طلب می‌كند.

Title: عفونت‌هاي شغلي پزشکان (پیش‌گیری و درمان)

http://hsr.mui.ac.ir/index.php/jhsr/article/view/25

پزشکان در هنگام مواجهه با بيماران، در خطر ابتلا به تعداد زيادي از عوامل عفوني هستند. با اين وجود با اعمال روش‌هاي ساده‌اي مانند واكسيناسيون، استفاده از دستكش، ماسك و شستن دست‌ها مي‌توانند خود را در مقابل بسياري از اين عوامل عفوني حفظ كنند. استفاده از روش‌هاي پيشرفته جراحي و استفاده صحيح از تكنيك‌هاي جراحي نيز خطر عوامل عفوني را كاهش مي‌دهد. در اين مقاله به مواردي اشاره خواهد شد كه با رعايت آن‌ها احتمال خطر براي پزشکان و بيمارانشان كاهش خواهد يافت.

Title: مروری بر تعارض در منافع در نگارش، بررسی و چاپ دستاوردهای دانشمندان علوم سلامت

http://hsr.mui.ac.ir/index.php/jhsr/article/view/19

تعارض در منافع، که اغلب در دو بعد اقتصادی و غیر اقتصادی ظهور می‌نماید، ممکن است نویسندگان، سردبیران، اعضای هیأت تحریریه و داوران را از انجام فعالیت اصلی خود، رعایت امانت و صداقت در امر نگارش، چاپ و انتشار دستاوردهای پژوهشی، باز دارد. در صورت رخ‌داد چنین امری، سلامت آحاد جامعه ممکن است در معرض خطر قرار گیرد. بنابراین، بهترین راه مقابله با این پدیده، شناخت و افشای صحیح و به موقع آن می‌باشد. هدف از نگارش مقاله مروری حاضر، آشنا ساختن دانشمندان فارسی زبان در حوزه سلامت با این پدیده و شیوه‌های مقابله با آن بود.
